# α-Santalol functionalized chitosan nanoparticles as efficient inhibitors of polo-like kinase in triple negative breast cancer

**DOI:** 10.1039/c9ra09084c

**Published:** 2020-02-03

**Authors:** Jinku Zhang, Yanan Wang, Jinmei Li, Wenming Zhao, Zhao Yang, Yanguang Feng

**Affiliations:** Department of Pathology, Baoding First Central Hospital Baoding 071000 Hebei province China zjkblk@sina.com; Department of Pathology, Affiliated Hospital of Hebei University Baoding 071000 Hebei province China; College of Life Science and Technology, Beijing University of Chemical Technology Beijing 010000 China; Department of Cardiology, Baoding Qingyuan District People's Hospital Baoding 071100 Hebei province China 13930832196fyg@sina.com

## Abstract

Polo-like kinase 1 (PLK-1) is a protein kinase that plays a significant role in the initiation, maintenance, and completion of mitotic processes in the cell cycle. PLK-1 has been recorded to be over-expressed in various human cancers and is associated with poor prediction; thus it is an attractive target for anticancer therapy. Novel α-santalol functionalized chitosan nanoparticles were synthesized using the sol gel method and were assessed for their *in vitro* (MTT, apoptotic staining assays, and cell cycle analysis) and *in vivo* activities. α-Santalol loaded chitosan NPs inhibited the proliferation of triple negative breast cancer (MDA-MB-231) at an inhibitory concentration of (IC_50_) about 4.5 μg mL; meanwhile, in normal cells, no adverse effects were exhibited up to 100 μg mL^−1^. The findings also implicated a decreased expression of the anti-apoptotic protein, BCL-2 with PLK-1 and an increase in the expression of BAD, caspases and BAX. However, in *in vivo* studies, the treated animal group exhibited no aberrant effects in vital organs or blood parameters. Tumor growth was significantly inhibited after i.v. injection of α-santalol loaded chitosan NPs at a dose of 5 mg kg^−1^. Taken together, the α-santalol functionalized chitosan NPs hold great potential in biomedical applications, especially cancer theranostics, due to their versatile nature as well as diagnostics for clinical tumor biology.

## Abstract 2

Polo-like kinase 1 (PLK-1) is a protein kinase that plays a significant role in the initiation, maintenance, and completion of mitotic processes in the cell cycle.

## Graphical abstract

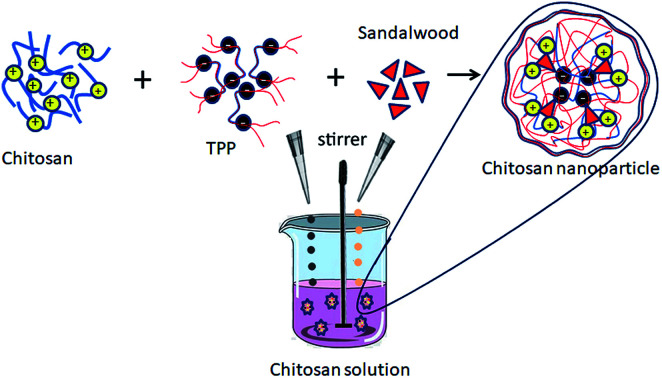

## 1. Introduction

Triple negative breast cancer (TNBC), a heterogeneous and aggressive cancer with a poor prognosis, comprises 15–20% of the overall breast cancer cases worldwide. The percentage of TNBC cases ranges from 6.7–27.9% in different countries and India has the highest rate amongst all of them with 27.9%, followed by Indonesia, Algeria, and Pakistan.^1^ The triple negative breast cancer cells lack of expression of estrogen (ER), progesterone (PR) and human epidermal growth factor 2 (HER 2) receptors on cell surface.^2^ TNBC has a propensity to propagate to various visceral organs including the liver and lung.^3^ Due to the absence of the receptors, the cancer cells do not react to either endocrine or HER-2 targeted therapies. Thus, the recent research findings by various researchers in treating TNBC relies on targeting it with PARP inhibitors, including the PD-L1 protein.^4^

One example involves the inhibition of polo-like kinase 1 (PLK-1), which pertains to the polo-like kinase family and is referred to as the master mitotic regulator.^5^ The PLK-1 gene is characterized by the presence of the canonical kinase catalytic domain with a regulatory domain of one or two polo-box domains (PBD).^6^ It is a pivotal gene responsible for the proper accomplishment of the cell division.^7^ PLK-1 is the responsible candidate gene and is highly synchronized with multi-layered regulation as it plays a pivotal role in the manifold stages of cell division including the entry of the cell into mitotic division,^8^ centrosome disjunction and movement, spindle formation,^9^ activation of anaphase by forming complexes,^10^ and cytokinesis.^11^ PLK-1 gene function is not only narrowed down to cell division but it is also responsible for DNA replication, transcription, translation, p53 regulation, and cell motility.^12–16^ PLK-1 expression is reported to be increased in various cancers including colon cancer, breast cancer, gastric cancer, and osteosarcoma and is embodied as an oncogene.^17,18^ For this reason, PLK-1 has served as a marker for various kinds of prognostic cancers^19^ and also has been used as a therapeutic target in various cancer studies.^20,21^

Thus, as a respite for TNBC, the PLK-1 gene was targeted with chitosan NPs. The NPs are nano-sized colloidal particles with a diameter of 10–500 nm, through which the bioactive compound can be loaded, adsorbed or conjugated.^22^ Chitosan (poly[-(1,4)-2-amino-2-deoxy-d-glucopyranose]), a naturally abundant bioactive polymer, formed by the deacetylation of chitin, is known for its myriad of properties including biodegradability, non-immunogenicity, biocompatibility, and non-toxicity.^23^ It is an FDA approved co-polymer consisting of glucosamine and *N*-acetyl glucosamine, which are cross-linked by a 1,4-glycosidic bond. Moreover, chitosan has the propensity to encapsulate or coat a drug over the surface and can also control the drug release proficiency.^24^ The polymer has been investigated as an adjuvant and for a wide variety of activities, including anti-microbial activity, anti-tumor activity, blood hemostatic activity, and anti-diabetic activity, as well as for enzyme immobilization and wound dressing applications.^25–28^ The mucoadhesive nature of chitosan raises the residual time at the site of absorption and its cationic property permits ionic linkages, which happens with multivalent anions.^29^

Different methods have been employed for synthesizing the chitosan NPs including ionic gelation using cross-linking agents (tripolyphosphate (TPP)^30^ or glutaraldehyde),^31^ the emulsion solvent evaporation method,^32^ and coacervation phase separation.^33^ In the present study, the chitosan NPs were synthesized using the ionic gelation method, where the NPs are formed by the interaction between positively charged chitosan chains (–NH_3_^+^) and the polyanion, which are used as cross-linkers. TPP and glutaraldehyde are the polyanions used. Owing to the reported toxic effects of glutaraldehyde,^34^ TPP is the chief polyanion used in the synthesis of chitosan NPs.

The synthesis of the chitosan particles is followed by the functionalization of the NP with α-santalol, a potent bioactive sesquiterpene compound present in the essential oil of *Santalum album* (East Indian sandalwood tree). The heartwood and the oil of *Santalum album* have been extremely valued for centuries. They are used as flavor constituents in the food industry and also used in the manufacture of cosmetics and perfume due to their fragrance.^35^ The major constituents of the essential oil of *Santalum album* comprise α-santalol and β-santalol,^36^ which deliver a myriad of activities including anti-oxidant activity, stress modulation effects,^36^ anti-hyperglycemic activity,^37^ anti-viral activity^38^ and also have neuroleptic effects.^37^ Additionally, they have potent anti-cancer activity against breast cancer,^39^ skin cancer,^40^ and prostate cancer by impeding angiogenesis and also targeting the vascular endothelial growth factor (VEGF) receptor-mediated signaling pathway.^41,42^

Hence, the present study was aimed at synthesizing and functionalizing chitosan NPs with α-santalol to target the activity of the polo-like kinase in triple negative breast cancer cells and *in vivo* in xenograft mice as a competent approach for treating TNBC.

## 2. Materials and methods

### 2.1. Materials

The phyto bioactive principle α-santalol was purchased from the Parchem Company private Ltd with a purity of 99%.

### 2.2. Method

#### 2.2.1. Synthesis of chitosan nanoparticles

**Scheme 1:**
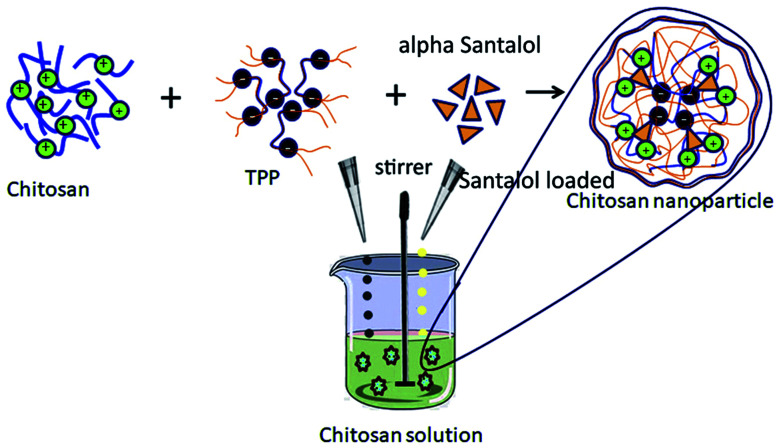
Schematic illustration of the synthesis of α-santalol loaded chitosan nanoparticles.

The α-santalol loaded chitosan NPs (Sn-CNPs) were fabricated using the ionotropic gelation technique reported by Elzatahry and Eldin in 2008, with slight modifications.^43^ The synthesis involved the addition of an aqueous solution of sodium TPP (18 mL, 1 mg mL^−1^) in a drop-wise manner into a solution of chitosan (35 mL, 1 mg mL^−1^) with and without α-santalol (10 mg mL; dissolved in 0.1% DMSO). The chitosan solution was made by dissolving 1% (v/v) acetic acid and continuously stirring. The pH of the solution was adjusted to 4.6 using 0.1 N NaOH. The NPs were molded without any aggregation following continuous stirring overnight. After overnight stirring, the chitosan nanoparticles and Sn-CNPs were subjected to centrifugation at 10 000 rpm at 4 °C. Subsequently, the supernatant was discarded, and the pellet was washed with 10% aqueous ethanol (Scheme 1) and preserved at 4 °C for further use.

#### 2.2.2. Loading and encapsulation efficiency of α-santalol

The Sn-CNPs were dispersed in phosphate buffer saline (PBS, 6 mL) and centrifuged at 12 000 rpm for 30 min. The supernatant was collected to measure the ultraviolet absorption at 280 nm. The loading efficiency and encapsulation efficiency of α-santalol in the chitosan NPs were calculated as follows:Loading efficiency = *W*_0_/*W* × 100%Encapsulation efficiency = *W*_0_/*W*_1_ × 100%where *W*_0_ is the amount of α-santalol loaded with the chitosan NPs, *W* is the amount of chitosan NPs, and *W*_1_ is the amount of chitosan added in the system.

#### 2.2.3. *In vitro* α-santalol release studies

The drug release profile of prepared Sn-CNPs was investigated at different pH, namely 5.5, 6.8, and 7.4. The α-santalol loaded chitosan nanoparticles were dispersed in PBS (pH 7.4) and transferred into a dialysis bag. The dialysis bag was immersed in PBS (95 mL) at pH 5.5, 6.8, and 7.4 with continuous stirring. The drug release profile was done at 37 °C. About 5 mL of the aqueous solution was withdrawn and substituted with 5 mL of fresh PBS at constant time intervals. After the experiment, the dialysate samples were evaluated by assessing the absorbance at 280 nm for α-santalol using UV-Vis spectroscopy.

#### 2.2.4. Characterization of functionalized chitosan nanoparticles with α-santalol

##### 2.2.4.1. UV-Vis spectral analysis

In order to confirm the formation of the NPs, the synthesized NPs were subjected to UV-Vis spectral analysis with the visible wavelengths covering a range from approximately 200 to 600 nm, using a quartz cuvette.

##### 2.2.4.2. FT-IR characterization

Fourier transform infrared (FT-IR) spectroscopy of the synthesized NPs was done with the help of a Nicolet 5700 instrument (Nicolet Instrument, Thermo Company, USA) by following the KBr pellet method. The synthesized NPs were ground with potassium bromide to form a translucent disc, which was then scanned over a wavelength range from 4000 cm^−1^ to 400 cm^−1^.

##### 2.2.4.3. Zeta potential analysis

The zeta potential of the synthesized chitosan NPs was determined by means of a zeta potential analyzer (90 Plus Particle Size Analyzer, Brookhaven Instruments Corporation, using Zeta plus software). The study was carried out at a scattering angle of 90° and a temperature of 25 °C with the NPs dispersed in deionized distilled water. The determination of the zeta potential is based on the direction and velocity of the particles under the impact of a known electric field.

##### 2.2.4.4. Dynamic light scattering (DLS) analysis

The particle size range of the synthesized chitosan NPs along with its distribution was determined using a particle size analyzer (90 Plus Particle Size Analyzer, Brookhaven Instruments Corporation). The particle size was determined based on measuring the time-dependent variation of laser light scattering by the NPs undergoing Brownian movement. The distribution of the synthesized NPs was given as a polydispersity index (PDI).

##### 2.2.4.5. Scanning electron microscopy (SEM) analysis

The synthesized chitosan NPs were placed on a polycarbonate substrate and the excess water was left to dry at room temperature. They were dried in a critical point dryer using carbon dioxide, sputter-coated with gold in a metallizer, and examined under a scanning electron microscope (JSM5600LV, JEOL, Japan) working at an accelerating voltage of 20 kV.

### 2.3. *In vitro* study

#### 2.3.1. Cell culture

The triple negative breast cancer cell line, MM2 (MDA-MB-231), was procured from the National Centre for Cell Sciences (NCCS), Pune, India. Subsequently, the cell line, MDA-MB-231, was maintained in Dulbecco's modified eagles medium (DMEM) supplemented with l-glutamine (2 mM) and balanced salt solution (BSS) adjusted to contain glucose (1.5 g L^−1^), Na_2_CO_3_ (1.5 g L^−1^), sodium pyruvate (1 Mm), non-essential amino acids (0.1 mM), l-glutamine (2 mM), (4-(2-hydroxyethyl)-1-piperazineethane sulfonic acid) (HEPES, 10 mM), and 10% fetal bovine serum (GIBCO, USA). Penicillin and streptomycin (100 IU/100 μg) were adjusted to 1 mL L^−1^. The cells were maintained at 5% CO_2_ with 37 °C.^44^

#### 2.3.2. Evaluation of the cytotoxicity of the synthesized nanomaterial

In order to evaluate the inhibitory concentration (IC_50_) value, an MTT (3-(4,5-dimethylthiazol-2-yl)-2,5-diphenyltetrazolium bromide, Hi-Media) assay was done. The MDA-MB-231 cells were grown (1 × 10^4^ cells per well) in a 96-well plate for 48 h to get 80% cell confluence. The media was substituted with fresh media containing α-santalol, chitosan NPs, and Sn-CNPs (10, 20, 30, 40, and 50 μg mL^−1^) and the cells were further incubated for 24 h. The culture media was removed. MTT solution (100 μL) was added to each well and the cells were incubated at 37 °C for 4 h. Then, the supernatant was removed. DMSO (50 μL) was added to each well and the cells were incubated for 10 min to solubilize the formazan crystals. Using an ELISA multiwell plate reader (Thermo Multiskan EX, USA), the optical density was measured at 570 nm and it was then used to calculate the percentage viability, which has been given by the following formula:^45^% viability = (OD value of experimental sample/OD value of experimental control) × 100

#### 2.3.3. Morphological study

The selected human MDA-MB-231 cancer cells were allowed to culture on a cover slip (1 × 10^5^ cells per cover slip) followed by incubation with α-santalol, chitosan NPs, and Sn-CNPs (IC_50_). They were fixed with an ethanol : acetic acid solution (3 : 1, v/v). Three monolayers per experimental group were micrographed. Further, the morphometric analysis was carried out by gently mounting the cover slips on the glass slides and focused using a Nikon (Japan) bright field inverted light microscope at 40× magnification.^45^

#### 2.3.4. Fluorescence microscopic analysis of apoptotic cell death

Approximately 1 μL of a dye mixture (100 mg mL^−1^ acridine orange (AO) and 100 mg mL^−1^ ethidium bromide (EtBr) in distilled water) was mixed with 0.9 mL of the cell suspension (1 × 10^5^ cells per mL) on clean microscope cover slips. The pretreated cancer cells were collected, washed with PBS (pH 7.2) and stained with the AO/EtBr solution (10 μL). After incubation for 2 min, the cells were washed twice with PBS (5 min each) and visualized under a fluorescence microscope (Nikon Eclipse, Inc, Japan) at 400× magnification with an excitation filter of 580 nm. Similarly, the cells were placed on glass coverslips in a 6-well plate and treated with α-santalol, chitosan NPs, and Sn-CNPs (IC_50_) for 24 h. The fixed cells were permeabilised with 0.2% Triton X-100 (50 μL) for 10 min at room temperature and incubated for 3 min with DAPI (10 μL). The coverslip was placed above the cells, which allowed for an even distribution of the stain. The cells were observed under a fluorescence microscope (Nikon Eclipse, Inc, Japan).^45^

#### 2.3.5. Detection of apoptosis by Annexin-V/FITC- flow cytometry

MDA-MB-231 cells (1 × 10^5^) were seeded in a 6-well plate. After 24 h incubation at 37 °C (5% CO_2_), the medium was changed with α-santalol, chitosan NPs, and Sn-CNPs (IC_50_). After 24 h incubation, the cells were harvested with trypsin, washed with PBS, and fixed in 70% ethanol. They were stored at −20 °C for 1 h. The cellular nuclear DNA was stained with annexin V-FITC as described, followed by incubation at 37 °C for 30 min. Flow cytometry was performed in duplicate with a BD FACS Verse flow cytometer. From each sample, 10 000 events were collected and fluorescence signal intensity was recorded and analyzed by CellQuest and Modifit.

### 2.4. Western blotting

Western blotting was performed to detect the regulation of apoptotic and anti-apoptotic proteins in treated cells. MDA-MB-231 cells (1.5 × 10^6^) were seeded onto 100 mm × 20 mm culture dishes in the presence of α-santalol, chitosan NPs, and Sn-CNPs (5, 10, 20 μg mL^−1^), and were treated for 24 h. The medium was removed and the cells were washed with PBS (0.01 M, pH 7.2) several times. Following the removal of the supernatant solution, the cells were lysed with lysis buffer (0.1 mL of lysis buffer for each plate) for 20 min. The supernatants were collected by centrifugation at 10 000 g for 5 min at 4 °C and were used as the cell protein extracts. The collected protein concentration was measured using a protein assay kit. The same amount of protein from each sample was applied to 12% SDS-polyacrylamide gel electrophoresis. Proteins were shifted onto a nitrocellulose membrane and then blocked for 1 h by means of 10% skimmed milk in water. After washing with PBS containing 0.1% Tween 20 for three times, primary antibodies against BAD, BAX, caspase 3, caspase 9, Bcl-2, β-actin, and PLK-1 were added at a v/v ratio of 1 : 1000. Subsequently, the samples were incubated overnight at 4 °C, and then the primary antibodies were washed. This was followed by the addition of the secondary antibodies after 1 h incubation at room temperature. Then the protein bands were analyzed.

### 2.5. RT-PCR

The MDA-MB-231 cells were treated in the presence of α-santalol, chitosan NPs, and Sn-CNPs (5, 10, 20 μg mL^−1^), and also an untreated control was set up. After 24 h of treatment, the media was removed. TriZol reagent was used to quarantine total RNA and it was reverse transcribed. The cDNA was isolated using the cDNA synthesis kit and amplified according to the instructions. RT-PCR analysis for quantifying the apoptotic genes was performed in a 20 μL reaction mixture containing random primer pairs (forward and reverse 0.5 μL + 0.5 μL) (1.0 μL), 10× reaction buffer containing a master mix (25 mM L^−1^ MgCl_2_, 10 mM L^−1^ dNTPs, Taq polymerase 2.5 U) (10 μL), cDNA as a template (2 μL) and the remaining volume was nuclease free dH_2_O (7 μL). Amplification cycles consisted of denaturation at 94 °C for 1 min, primer annealing at 55 °C for 40 seconds and extension at 72 °C or 1 min, for a total of 32 cycles followed by a final extension at 72 °C for 10 min.

### 2.6. *In vivo* study

#### 2.6.1. *In vivo* assessment of anti-tumor activity

This study was performed in strict accordance with the NIH guidelines for the care and use of laboratory animals (NIH Publication No. 85-23 Rev. 1985), and was approved by the Institutional Animal Care and Use Committee of Bharathiar University (IAEC, 722/Go/Re/S/02/CPCSEA – India). Additionally, in this study, no informed consent human subjects were used. *In vivo* acute toxicity was assessed with young and healthy, non-pregnant, nulliparous swiss albino mice, weighing about 25–28 g (8 weeks old), which were placed in clean polypropylene cages with access to food and water by following OECD guidelines (OECD, 2001). These cages were placed in an air-conditioned animal house with a relative humidity of about 65–80% and at a temperature of 26–28 °C with 12 h light–dark cycles. The animals were acclimatized for one week by feeding with mice pellets, which are commercially available, and they were also given deionized water.

The efficacy of the compound against the tumor was assessed by implanting the tumor in the mice. This can be done by introducing the MDA-MB-231 cells [1 × 10^7^ in 100 mL of normal saline] to the subcutaneous dorsa of the mice. When the size of the xenograft tumor reached about 40–60 mm^3^, the mice were randomly segregated into 5 groups with 5 mice in each group: Group 1- mice treated with PBS (negative control), Group 2- mice treated with Doxorubicin (positive control), Group 3- mice treated with chitosan NPs (10 mg mL^−1^), Group 4- mice treated with α-santalol (8 mg mL^−1^), Group 5- mice treated with Sn-CNPs (5 mg mL^−1^). The agents were administered to the mice through intravenous injection and the mice were then observed for 21 days. At three day intervals, the diameters of the tumors were measured for each animal group. The body weight and tumor volumes (*V*) were calculated using the formula *V* = length × (width)^2^/2. Histopathological analysis (brain, heart, kidney, lung, and liver) was done following animal sacrifice by cerebral dislocation. Tissue slides were prepared and stained with hematoxylin and eosin stain (H and E stain). Further, they were visualized under a light microscope.

#### 2.6.2. Hematological and biochemical parameters

The whole blood was centrifuged at 3000 rpm for 15 min in order to obtain the serum. Serum biochemical levels including total cholesterol, total protein (TP), alkaline phosphatase (ALP), uric acid, tri-glyceride (TG), blood urea nitrogen (BUN), serum glutamic oxaloacetic acid (SGOT), and serum glutamic pyruvic transaminase (SGPT) were examined. 0.1 mL of 15 g L^−1^ EDTA-Na was pre-added into a 1 mL blood sample and the blood sample was used for the blood element test within 2 h. The blood-elements, including total count of red blood cells (RBCs), white blood cells (TC), dendritic cells (neutrophils, lymphocytes, monocytes, eosinophils, and basophils), and blood platelets were assayed.

#### 2.6.3. Hemolytic assay

Hemolytic assays were done on the collected blood, which was maintained using the stabilizing agent, ethylenediaminetetraacetic acid (EDTA). To PBS (8 mL), blood (4 mL) was added. This was followed by centrifugation at 10 000 rpm for 5 min in order to isolate the RBCs and further washing five times with sterile PBS solution. Then the RBCs were diluted with PBS (40 mL). Subsequently, the diluted RBC suspension (0.2 mL) was added to a concentration of 5, 10 or 20 μg mL^−1^ of α-santalol, chitosan NPs, and Sn-CNPs, respectively. The sample vials were kept without disturbance for 3 h at room temperature. Finally, the vials were centrifuged at 10 000 rpm for 3 min and 100 μL of supernatant from all samples were taken. Absorbance was noticed at 543 nm. The percentage of hemolytic activity was calculated using the following formula:
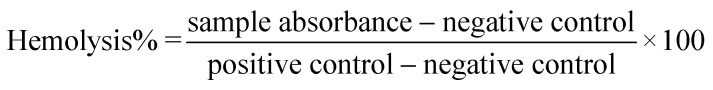
Here, the deionized water and PBS with RBCs were used as positive and negative controls.

### 2.7. Statistical analysis

The data was analyzed using the SPSS software. The results are presented as mean ± SD. Differences among various groups were evaluated by conducting one-way analysis of variance (ANOVA); *P* < 0.05 were considered statistically significant groups.

## 3. Result and discussion

### 3.1. Characterization of synthesized nanoparticle

#### 3.1.1. UV-Vis spectra and FTIR analysis

**Fig. 1:**
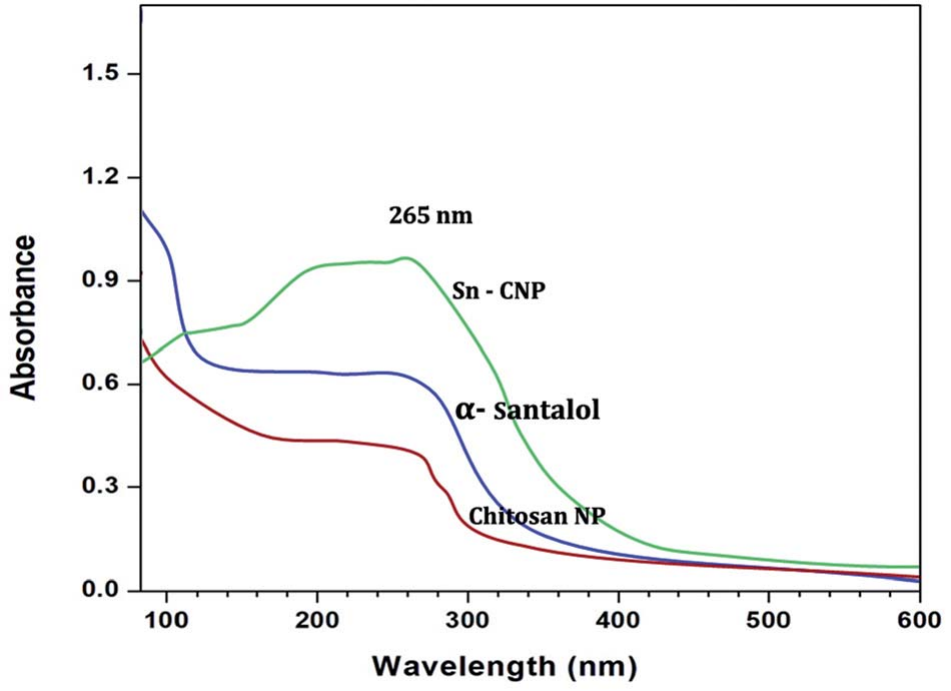
UV-Vis spectral analysis of the synthesized nanomaterials.

**Fig. 2:**
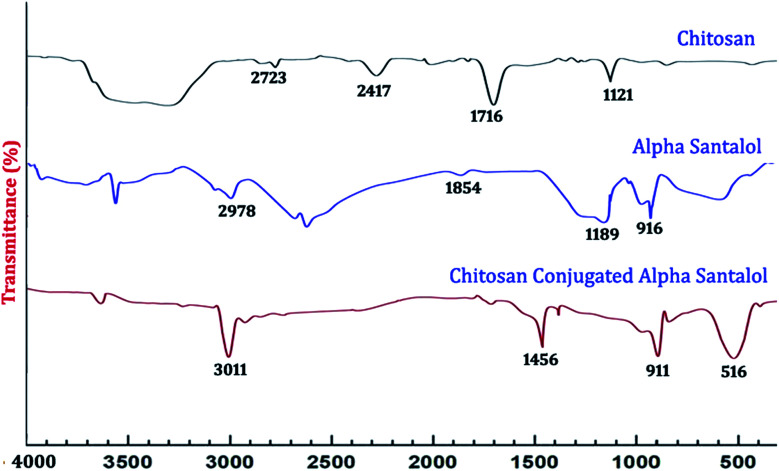
FT-IR spectral analysis of the synthesized nanomaterials.

The UV-Vis spectra of the synthesized nanoparticles are depicted in Fig. 1. The absorption peaks of chitosan NPs, α-santalol and Sn-CNPs were found to be at 265, 261, and 260 nm, respectively. The specific absorption peaks may be due to the presence of amide groups in the particles. Krishnaveni *et al.* in 2014^46^ had obtained an absorption signal at 310 nm for a chitosan NP while Biswapriya *et al.* reported the spectrum of α-santalol with a signal at 226 nm. The FTIR spectrum of the synthesized NPs is presented in Fig. 2 and depicts the organic functional group present on the surface of the NPs. The peaks of chitosan NPs at 2723 cm^−1^, 2417 cm^−1^, and 1716 cm^−1^ are recognized as stretching vibrations of C–H alkanes and the bending vibration at 1121 cm^−1^ is the fingerprint peak of chitosan, while the peak at 3375 cm^−1^ corresponds to NH_2_ and OH stretching vibrations.^47,48^ The α-santalol has peaks at 2978 cm^−1^ and 1854 cm^−1^, which depict the presence of O–H groups, C=H groups, and aromatic rings. The synthesized Sn CNP shows the corresponding peaks of 576 cm^−1^, 911 cm^−1^ represent the santalol coated particles remains have its bending vibrations with both the chitosan NPs and α-santalol.

#### 3.1.2. DLS and zeta potential analysis

**Fig. 3:**
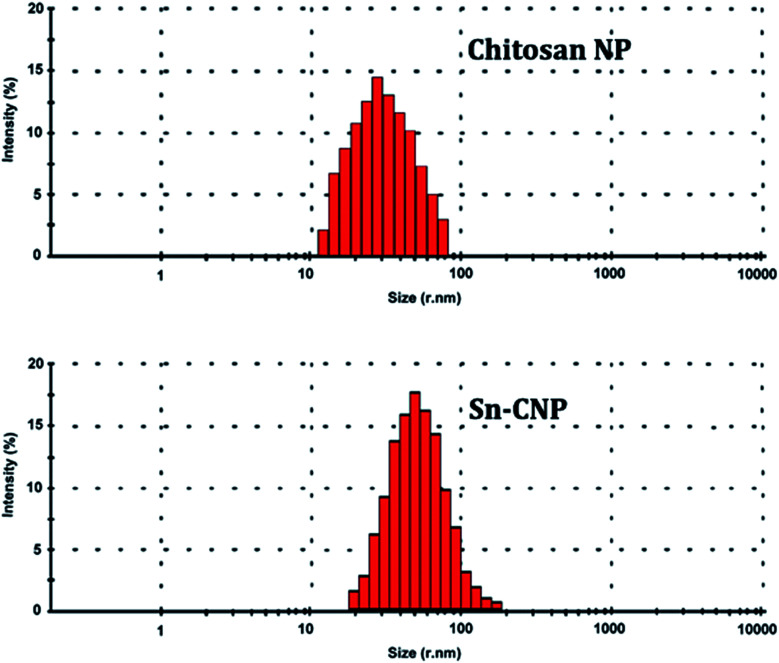
Dynamic light scattering analysis of the synthesized nanomaterials.

**Fig. 4:**
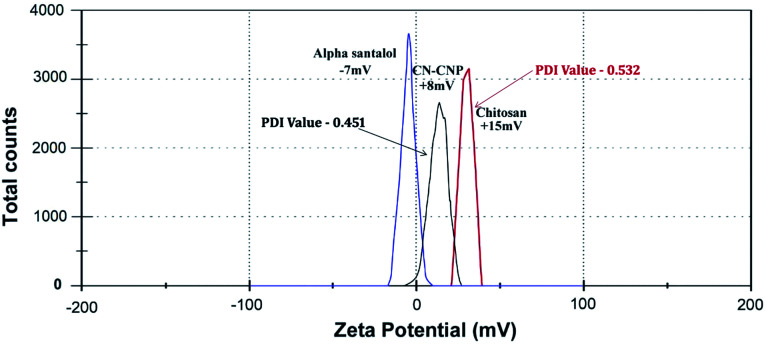
Zeta potential analysis of the nanomaterials.

The hydrodynamic diameter and surface charge of the synthesized NPs have a vital role in particle characterization and can be measured by DLS and a zeta sizer (Fig. 3 and 4). They give the dispersity, size, and charge of the NP. The size of the synthesized chitosan NPs and Sn-CNPs, shown in Fig. 3, were found to be 30 nm and 45 nm, respectively. The polydispersity of the synthesized NPs was also noted. The average size of the NP largely depends on the concentration of the chitosan and TPP used during the production process. The results obtained clearly state that the zeta potential of the synthesized Sn-CNPs was found to be +8 mV. In terms of dispersity index, the synthesized biomaterials are highly polydispersed in nature with multiple particle size populations. The polydispersion index values of the materials were determined to be about 0.451 for chitosan and 0.532 for Sn-CNPs, respectively. The higher positive charge of the potential indicates that the NPs are highly stable.^49^ A report by Blanco *et al.*^50^ highlighted that positively charged NPs can effectively target the tumor environment when compared to NPs with other net charges.

#### 3.1.3. SEM analysis

**Fig. 5:**
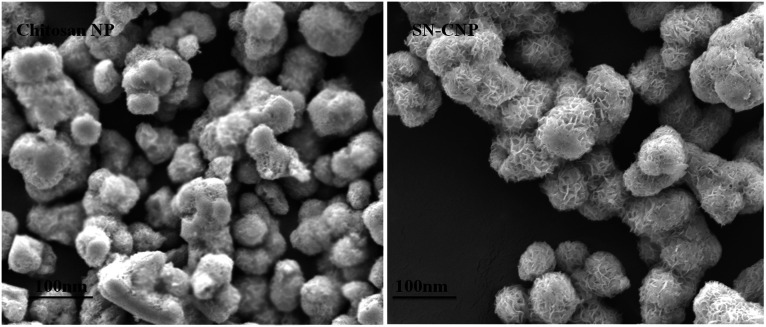
Scanning electron microscopic analysis of chitosan NPs and α-santalol loaded chitosan NPs.

The SEM images highlight the size and morphology of the synthesized NPs. The size of the synthesized chitosan NPs and Sn-CNPs were found to be 30 nm and 45 nm, respectively, while the particles were found to be spherical in shape (Fig. 5). The feathery appearance of the particles may be due to the firm conjugation of the santalol molecule on the surface of the chitosan NPs. Since the size of the NPs were found to be less than 100 nm, they have a greater capacity for accumulating in the tumor environment.^51^

#### 3.1.4. Loading and encapsulation efficiency of α-santalol

The loading and encapsulation efficiency of α-santalol in the chitosan NPs is depicted in Table 1. α-Santalol at three different concentrations (1, 4, and 8 μg mL^−1^) was used in the study. The loading and encapsulation efficiency is based on the highest concentration of 8 μg mL^−1^ with values of 16.7% and 72%, respectively.

**Table 1:** Loading efficiency and encapsulation efficiency of α-santalol functionalized chitosan nanoparticles

Parameter	α-Santalol concentration		
	1 μg mL^−1^	4 μg mL^−1^	8 μg mL^−1^
Loading efficiency (%)	6.5 ± 0.2	8.5 ± 0.5	16.7 ± 0.8
Entrapment efficiency (%)	22 ± 0.6	46 ± 0.2	72 ± 0.4

#### 3.1.5. Drug release profile of Sn-CNPs

**Fig. 6:**
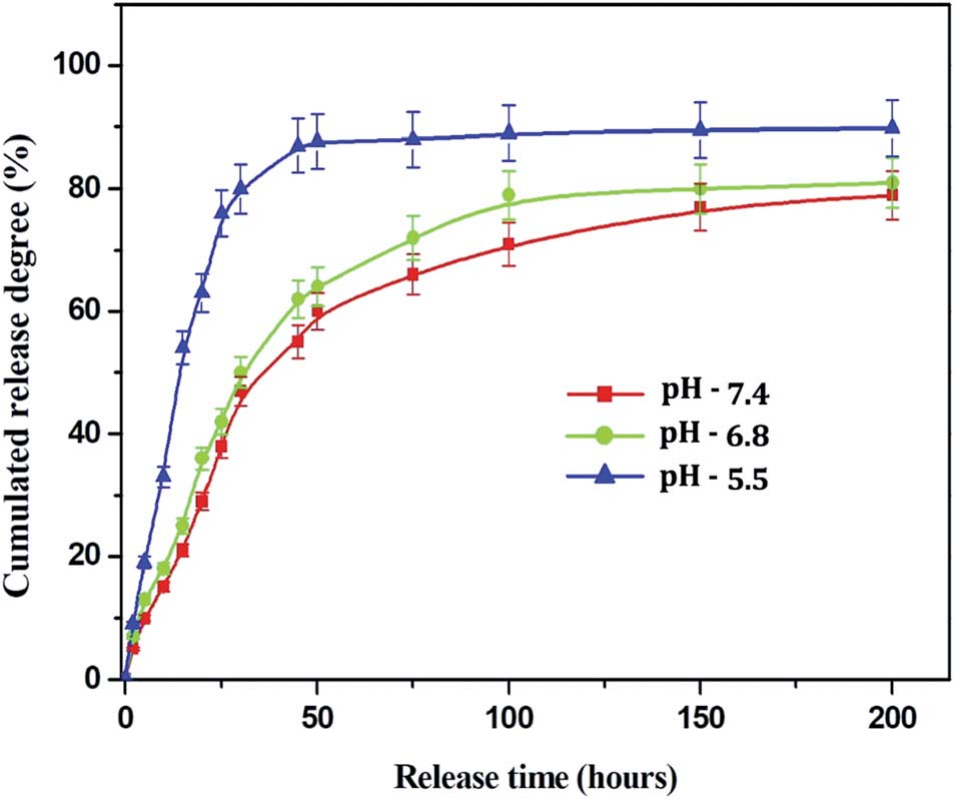
Drug release profile of the Sn-CNPs at different pH.

One of the most substantial characteristics of a drug delivery vehicle is its ability to release the payload drug efficiently into target cells. The drug release profile of the Sn-CNPs was recorded from 0 h up to 200 h under different pH conditions, namely pH 7.4 (the pH of blood), pH 6.8 (the pH of cancer cells), and pH 5.5 (the pH in the tumor microenvironment). An *in vitro* α-santalol release profile of the Sn-CNPs in PBS is shown in Fig. 6. The release rate decreased with increasing pH values. The results clearly demonstrated that Sn-CNPs revealed pH-responsive release behavior. Approximately 90% of the combined α-santalol was released from the loaded chitosan NPs in PBS at pH 5.5, at the maximum period of 200 h. At pH 6.8, the drug release ratio was slightly lower than that of pH 5.5. However, a prompt release happened at pH 7.4, where more than 70% of the entrapped α-santalol was released after an incubation period, which may be attributed to the acidic pH-induced deformation of the linkage between α-santalol and the chitosan NPs. It is well known that the extracellular pH of tumors is slightly more acidic than that of blood and normal tissues. Thus, the electrostatic interaction that exists in neutral surroundings will disappear in an acidic environment. Thus, pH 5.5 facilitates the efficient release of loaded α-santalol, which is significant as it implies that there will be better drug delivery in the tumor microenvironment.

### 3.2. *In vitro* analysis

#### 3.2.1. Effect of Sn-CNP on cell viability

**Fig. 7:**
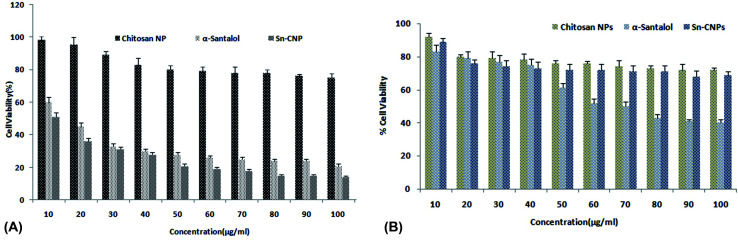
(A) Cytotoxic analysis of the synthesized nanomaterials against triple negative breast cancer cells (MDA MB-231). (B) Cytotoxic analysis of the synthesized nanomaterials against normal breast cells (MCF-10).

The cytotoxic activity of the functionalized chitosan NPs was analyzed. The compound showed a reduced percentage viability of the cells with increasing concentration of the nanomaterials. The *in vitro* cytotoxic effect of the NPs against MDA-MB-231 cells is depicted in Fig. 7A. Table 2 presents the analyzed inhibitory concentration (IC_50_) of the synthesized nanomaterials and it was found to be 13 μg mL^−1^ for α-santalol, 5 μg mL^−1^ for Sn-CNPs and insignificant toxicity was found up to 100 μg mL^−1^ for chitosan NPs. The IC_50_ concentration of the Sn-CNPs was found to be significantly lower when compared with that of the other treatment groups, whereas the synthesized nanomaterials were found to exhibit biocompatibility with normal breast cells (Fig. 7B). Santha *et al.* reported the anti-cancer efficacy of α-santalol against MDA-MB-231 cells to be higher with increasing concentration and with an elapsed period of treatment. Against normal breast cancer cells, the compound exhibited significant cell death only at high concentrations.^39^ Also, another study by Bommerady *et al.* reported that α-santalol showed low toxicity against normal prostate epithelial cells (PrEC), while it had a significant toxic effect against prostate cancer cells (PC-3).^41^

**Table 2:** Cytotoxic activity of the synthesized nanomaterials (μg mL^−1^)

Cytotoxic activity of sample (μg mL^−1^)		
Nanomaterials	MDA MB 231 (triple negative breast cancer)	MCF-10A (normal breast cells)
Chitosan NPs	Insignificant toxicity up to 80 μg mL^−1^	Insignificant toxicity up to 80 μg mL^−1^
α-Santalol	15 ± 1.0	Insignificant toxicity up to 70 μg mL^−1^
Sn-CNP	8.0 ± 0.7	Insignificant toxicity up to 80 μg mL^−1^

#### 3.2.2. Effect of Sn-CNPs on cell morphology

**Fig. 8:**
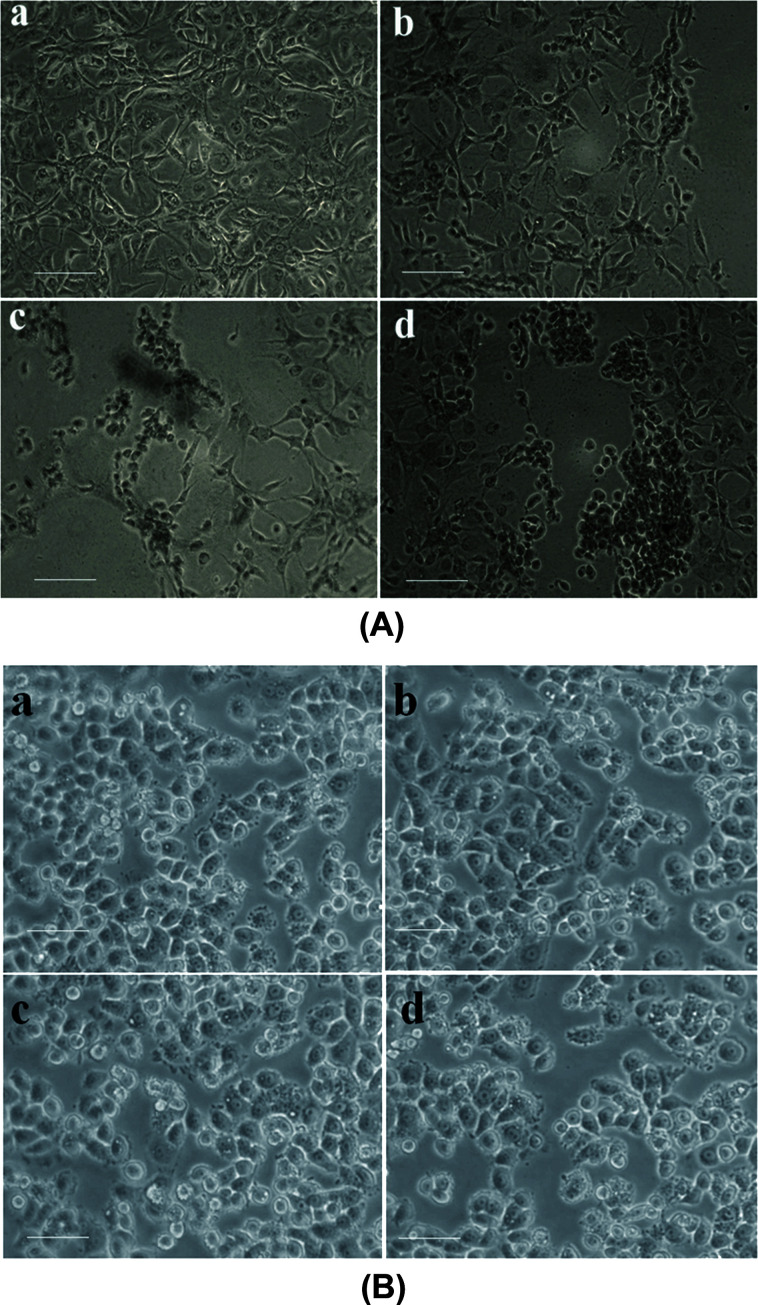
(A) Morphometric analysis of the synthesized nanomaterials on MDA-MB-231 cells under an inverted microscope (a) control (b) 100 μg mL^−1^ of chitosan NPs (c) 13 μg mL^−1^ of α-santalol (d) 5 μg mL^−1^ of Sn-CNP treated cells. The scale bar measures about 100 μm. (B) Morphometric analysis of the synthesized nanomaterials on MCF-10A cells under an inverted microscope (a) control; (b) 100 μg mL^−1^ of chitosan NPs; (c) 50 μg mL^−1^ of α-santalol; (d) 100 μg mL^−1^ of Sn-CNP treated cells. The scale bar measures about 100 μm.

The alterations which are made in the MM2 cells on account of treating it with the synthesized NPs at IC_(50)_ were examined and interpreted in Fig. 8A. It can be observed from the figure that the Sn-CNPs treated cells had shown significant morphological changes including membrane blebbing and cell shrinkage. Floating cells are also observed in a dose-dependent manner. In normal breast tissue, there was no significant toxic effect up to 100 μg mL^−1^ (Fig. 8B). It is a well-recognised phenomenon that the cytological examinations interpret the antiproliferative effect routed with the above mentioned changes through the synthesized nanoparticle. Thus, the treated cells showed distinguished morphology including fragmented apoptotic bodies, margination, and shrunken nuclei when compared to untreated cells, which are signs of apoptosis.^52^

### 3.3. Effect of Sn-CNPs on apoptosis and nuclear fragmentation

**Fig. 9:**
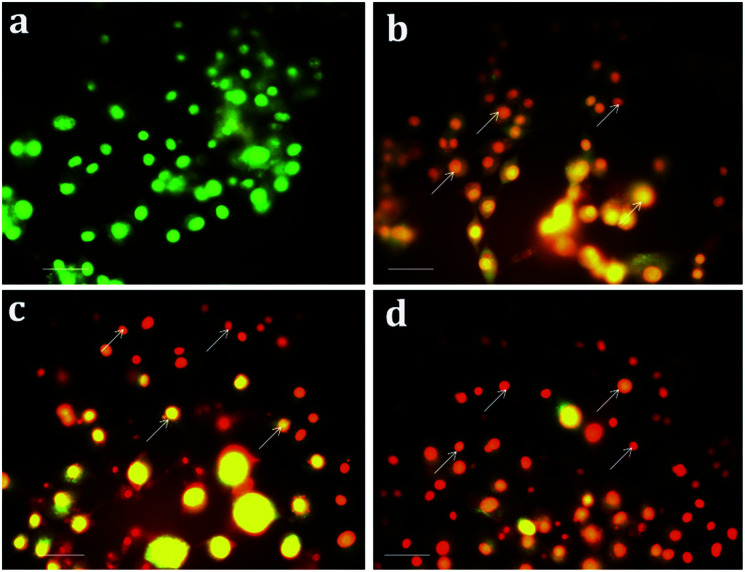
Apoptotic analysis of the synthesized nanomaterials on MDA-MB-231 cells under a fluorescence microscope (a) control; (b) 100 μg mL^−1^ of chitosan NPs; (c) 13 μg mL^−1^ of α-santalol; (d) 5 μg mL^−1^ of Sn-CNP treated cells. The scale bar measures about 100 μm and the white arrows indicate apoptotic bodies.

Apoptosis, a vital cellular pathway, plays an important role in maintaining the equilibrium of our body.^53^ In order to assess the apoptogenic activity of the synthesized NPs, fluorescence microscopic analysis was done using AO/EtBr staining. The fluorescence microscopic analysis of the chitosan NPs, α-santalol and Sn-CNP treated and untreated cancer cells are depicted in Fig. 9. Fig. 9a shows that the untreated MDA-MB-231 cells (control) did not have any significant effect when compared to the treated cells. Subsequently, Fig. 9b–d indicates the conversion of green-colored cells to orange/red colored cells, which is due to the initiation of apoptosis and the nuclear condensation effect occurred due to the action of the synthesized NPs.

**Fig. 10:**
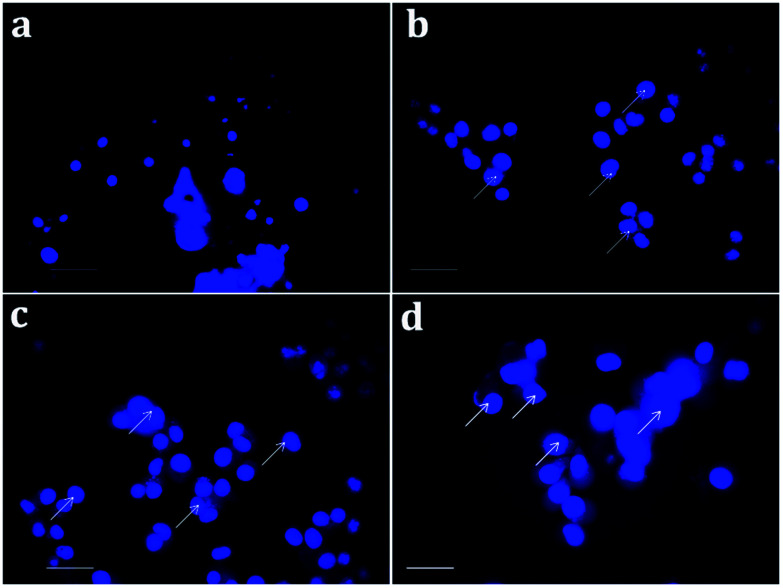
Nuclear fragmentation analysis of the synthesized nanomaterials on MDA-MB-231 cells (a) control; (b) 100 μg mL^−1^ of chitosan NPs; (c) 13 μg mL^−1^ of α-santalol (d) 5 μg mL^−1^ of Sn-CNP treated cells. The scale bar measures about 100 μm and the white arrows indicate nuclear fragmented cells.

In order to further distinguish the apoptotic nuclei from the healthy ones, 4′,6-diamidino-2-phenylindole (DAPI), a nuclear stain, was used and the results of the MDA-MB-231 cells (treated and untreated) are portrayed in Fig. 10. As seen in Fig. 10a, the untreated cells show weak fluorescence as they have intact round nuclei. In contrast, the functionalized NP treated cells (Fig. 10d) emit high fluorescence when viewed under a microscope, which is due to the nuclear fragmentation of condensed apoptotic nuclei. Similarly, Bommareddy *et al.* highlighted the effect of α-santalol on prostate cancer by apoptotic induction of the compound in PC-3 and LNCaP cells.^41^

### 3.4. Effect of Sn-CNP on apoptosis by flow cytometry

**Fig. 11:**
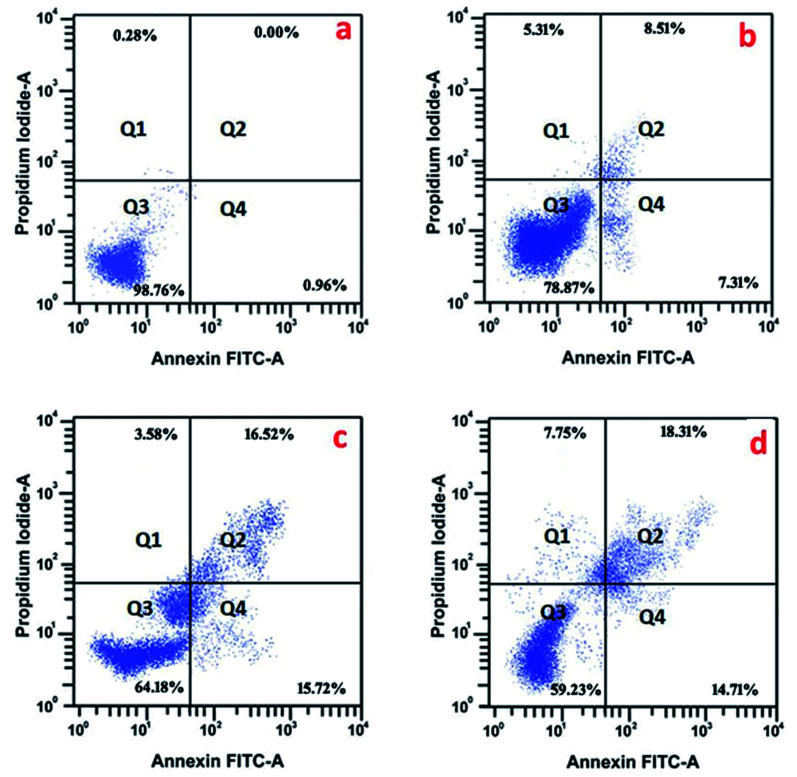
Apoptotic analysis of the synthesized nanomaterials by flow cytometry on MDA-MB-231 cells; (a) control; (b) 100 μg mL^−1^ of chitosan NPs; (c) 13 μg mL^−1^ of α-santalol; (d) 5 μg mL^−1^ of Sn-CNP treated cells. In the panel I, II, III, and IV refer to necrotic cells, late apoptotic cells, pro-apoptotic cells, and live cells, respectively.

In order to evaluate the mechanism by which the synthesized NPs exerted apoptosis, the cells were treated with Annexin V/FITC. The histogram (Fig. 11) of the cell cycle analysis describes the distribution of necrotic cells in the upper left quadrant (I); late apoptotic cells in the upper right quadrant (II); live cells in the lower left quadrant (III); and early apoptotic cells in the lower right quadrant (IV). From Fig. 11, it is found that the functionalized NPs increase the level of apoptotic cells in a significant way, evidencing their efficacy against TNBC. Kaur *et al.* noted strong apoptotic activity against skin cancer cells (A431) induced by α-santalol, which relates to the significance of the present study.^54^ As chitosan is a biocompatible polymer, it is not involved in the apoptotic cell death of the cancer cell,^55^ while the Sn-CNPs induced significant apoptotic death upon treatment.

### 3.5. Effect of Sn-CNPs on apoptotic protein expression

**Fig. 12:**
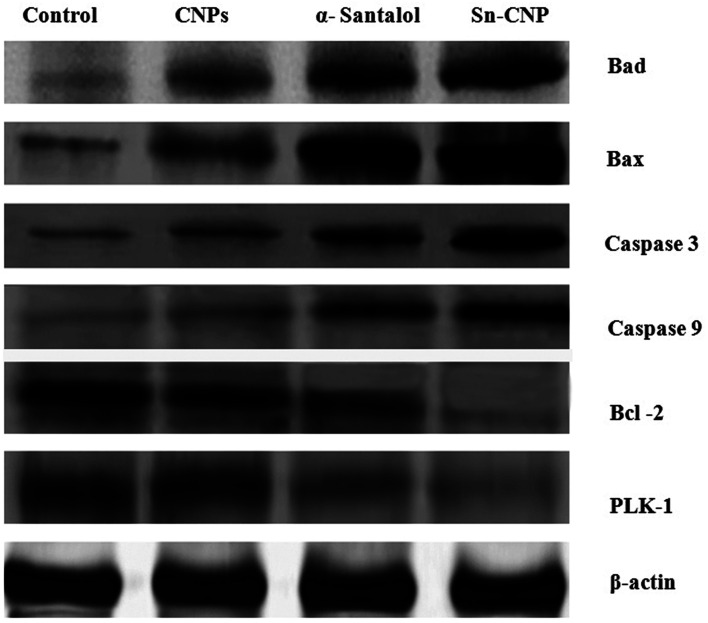
Expression of apoptotic and anti-apoptotic proteins by western blotting in MDA-MB-231 cells treated with the synthesized nanomaterials.

The signaling pathway of apoptosis involves a complex number of molecules.^56^ To disclose the apoptotic pathway induced by the Sn-CNPs in MDA-MB-231 cells, western blot analysis was done to quantify the expression of a wide range of apoptotic genes (Fig. 12). Remarkably, it was identified that there is an upregulation in apoptotic proteins including BAX, BAD, caspase-3, and caspase-9, while on the other hand there was a noticeable down-regulation in the anti-apoptotic protein BCL-2 and PLK-1. Notably, our results were correlated with the fact that depletion or down-regulation of PLK-1 will induce mitotic failure, DNA damage responses and finally apoptosis of the cells.^57^

### 3.6. Effect of Sn-CNPs on apoptotic gene expression

**Fig. 13:**
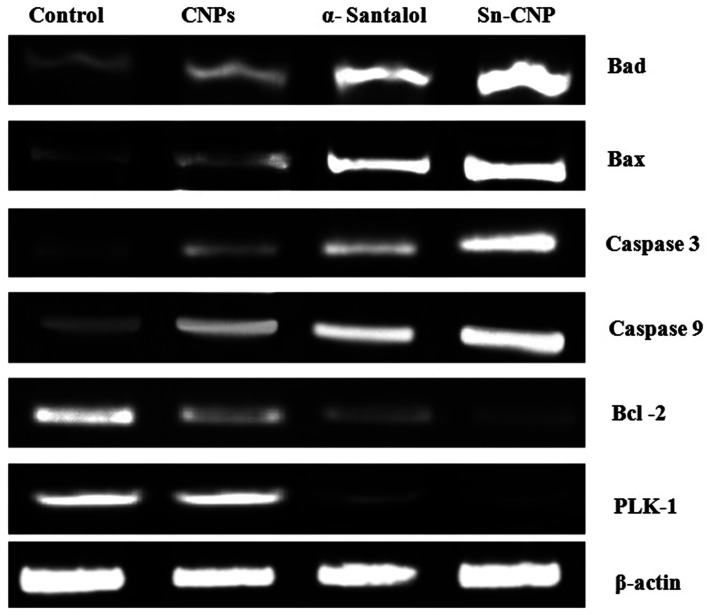
Expression of apoptotic and anti-apoptotic genes by RT-PCR analysis in MDA-MB-231 cells treated with the synthesized nanomaterials.

To investigate the targetability of the Sn-CNPs on PLK-1 gene expression in MM2 tumor cells, the RNA was quarantined from the treated cells and was subjected to RT-PCR. Our study (from Fig. 13) highlighted that the Sn-CNPs lower or down regulate the expression of PLK-1 in a significant way. Likewise, a previous study emphasized the activity of ASO-loaded HSA NPs on PLK-1 expression in treated BT-474 cells, which also demonstrated significant down-regulation.^58^

### 3.7. Evaluation of *in vivo* analysis of α-santalol loaded chitosan nanoparticle

**Fig. 14:**
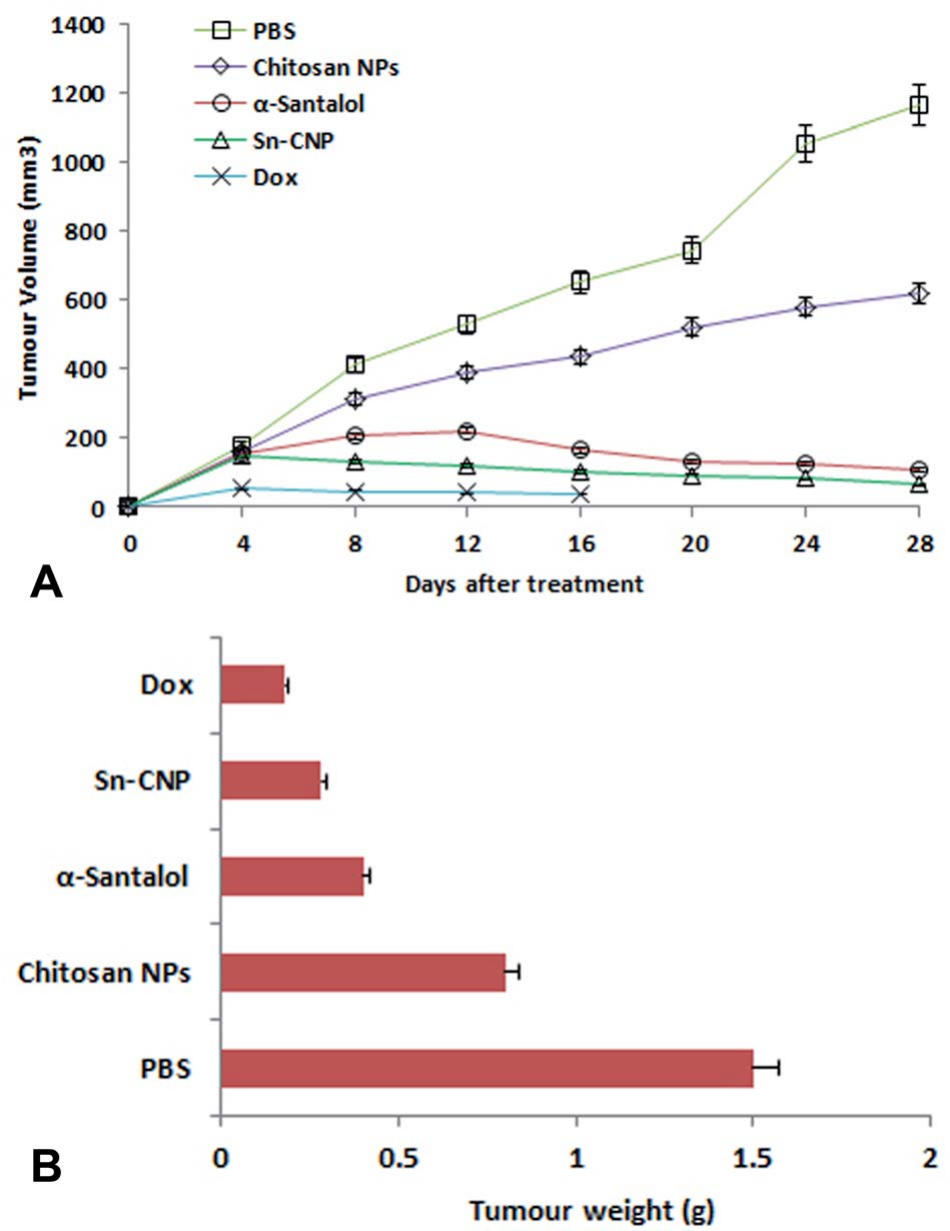
(A) Effect of the synthesized nanomaterials on tumor volume. (B) Effect of the synthesized nanomaterials on tumor weight.

**Fig. 15:**
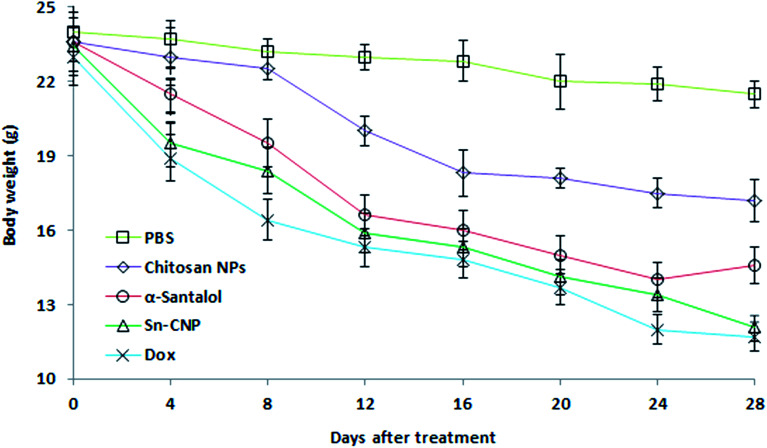
Effect of the synthesized nanomaterials on the body weight of the mice.

**Fig. 16:**
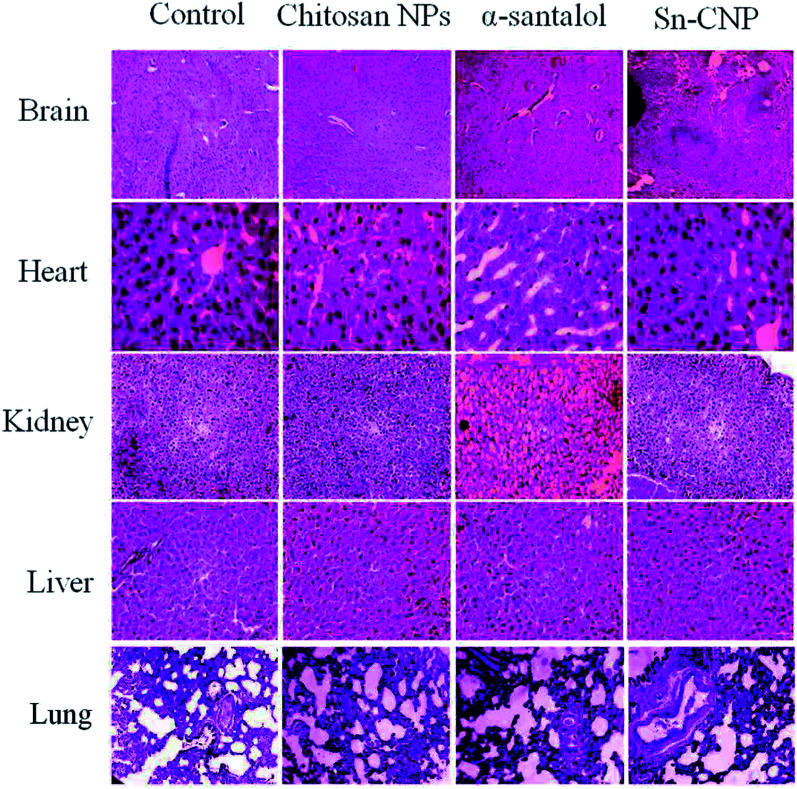
Histopathological analysis of the treated mice groups with the synthesized nanomaterials.

The efficacy of Sn-CNPs in *in vivo* therapeutics plays a pivotal role in determining their medical prospectives. The upshot of the synthesized NPs on the tumor volume and the body weight of the animals were examined and are shown in Fig. 14 and 15, respectively. There is a significant inhibition in the proliferation of MDA-MB-231 cells in the Sn-CNP treated mice, as denoted by the decrease in tumor volume (Fig. 14A) and tumor weight (Fig. 14B) when compared with the mice treated with saline. The Dox treated mice showed a significant reduction in tumor volume when compared with other treatment groups. Similarly, the mice administered with Sn-CNPs showed a significant reduction in their body weight when compared with the control group (Fig. 15). The histopathological analysis of H&E stained organs (brain, heart, kidney, lung, and liver) of mice treated with the Sn-CNPs showed no adverse effects at the desired concentration and are depicted in Fig. 16. The results of previous investigations highlighted that α-santalol is a potent inhibitor of skin papilloma proliferation in both CD-1 and SENCAR strains of mice.^59^

The hematological and biochemical parameters of animals from various treatment groups were analyzed and tabulated (Table 3). There is a decrease in hemoglobin, RBCs, and platelet counts when compared with that of the control group, while there was a differential count of WBC's. The monocyte count was increased whereas the neutrophil, lymphocyte, eosinophil, and basophil count was reduced. The biochemical parameters including cholesterol, uric acid, triglyceride, and SGPT levels increased whereas the alkaline phosphatase, total protein level, BUN, and SGOT levels decreased.

**Table 3:** A comparison of the hematological and biochemical parameters of the treated mice groups

Parameter	Control	α-Santalol	Chitosan NPs	Sn-CNP
RBC (million per mm^3^)	5.1 ± 0.5	4.95 ± 0.3	4.8 ± 0.2	4.75 ± 0.7
Hemoglobin (g dL^−1^)	13.3 ± 0.2	13.05 ± 0.4	12.96 ± 0.8	12.8 ± 0.2
WBC (T mm^−3^)	4.8 ± 0.4	4.3 ± 0.4	4.1 ± 0.7	3.9 ± 0.8
Neutrophils	35 ± 0.5	33 ± 0.2	32 ± 0.4	31 ± 0.7
Monocytes	3.3 ± 0.2	3.5 ± 0.7	3.6 ± 01	3.8 ± 0.5
Lymphocytes	55 ± 0.5	54 ± 0.2	53 ± 0.4	51 ± 0.8
Eosinophils	0.2 ± 0.5	0.2 ± 0.1	0.2 ± 0.3	0.1 ± 0.7
Basophils	0.3 ± 0.01	0.2 ± 0.05	0.2 ± 0.03	0.2 ± 0.05
Platelets (lakh per mm^3^)	1.5 ± 0.3	1.5 ± 0.2	1.49 ± 0.1	1.4 ± 0.7
Cholesterol (mg dL^−1^)	113 ± 0.7	115 ± 0.2	118 ± 0.3	119 ± 0.5
Uric acid (mg dL^−1^)	5.8 ± 0.1	6.0 ± 0.2	6.1 ± 0.8	6.3 ± 0.2
Alkaline phosphate (IU L^−1^)	61.5 ± 0.5	60.1 ± 0.1	59.4 ± 0.7	59.0 ± 0.6
Total protein (gm dL^−1^)	5.9 ± 0.5	5.84 ± 0.7	5.61 ± 0.4	5.3 ± 0.2
Triglyceride (mg dL^−1^)	51 ± 0.5	53 ± 0.4	56 ± 0.6	58 ± 0.1
BUN (mg dL^−1^)	12.0 ± 0.1	11.8 ± 0.3	11.4 ± 0.2	11.0 ± 0.5
SGPT (IU L^−1^)	12.1 ± 0.7	12.5 ± 0.8	12.8 ± 0.2	12.9 ± 0.1
SGOT (IU L^−1^)	16.5 ± 0.2	16.1 ± 0.5	15.8 ± 0.1	15.4 ± 0.7

**Fig. 17:**
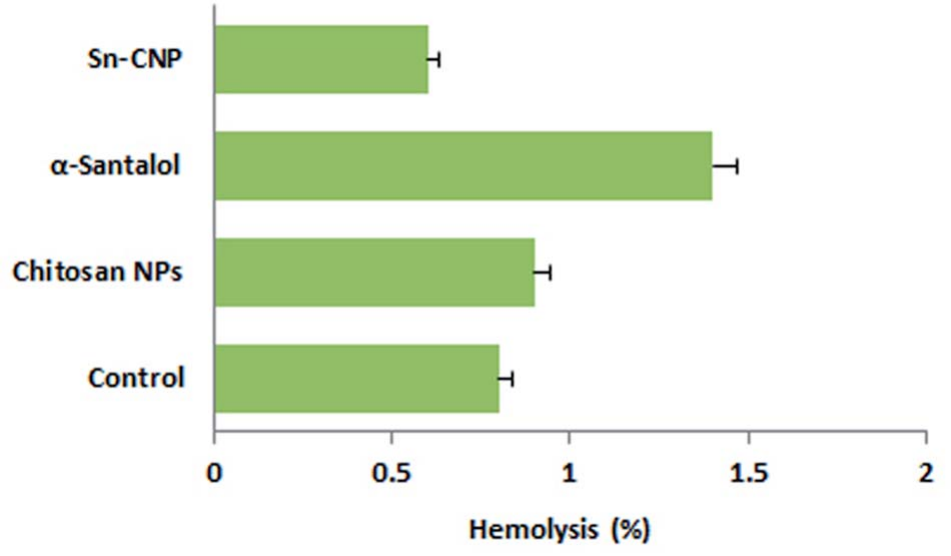
Hemolytic activity of the synthesized Sn-CNPs.

The hemocompatibility of the synthesized NPs with erythrocytes plays a pivotal role in the application of NPs in the biological system.^60^ The RBCs were exposed to synthesized nanomaterials, which were prepared for nearly 3 h. The hemolytic activity of the control, chitosan NPs, α-santalol, and Sn-CNPs were determined by measuring the release of the erythrocytes and are shown in Fig. 17. Based on a previous report, which highlighted the use of biomaterials until 5% hemolysis is observed,^61^ the SN-CNPs can be used as a potent biocompatible nanotarget to treat TNBC.

## 4. Conclusion

In this study, our findings nurture the opportunity of using phytocompound (α-santalol) as a targeting mediator against triple negative breast cancer both *in vitro* and *in vivo*. It can be used as an effective drug delivery system and also provokes a cytotoxic effect in selective triple negative breast cancer cells. Moreover, the Sn-CNPs were less toxic to normal breast cells and thus, assumed to be biocompatible in living systems at the given concentration. Additionally, it has the capability to initiate apoptosis inducing activities in the selected TNBC cells and at the same time, it can down-regulate the main cancer-promoting protein (PLK), leading to cell growth inhibitory activity. Taken as a whole, our synthesized nanomaterials effectively inhibit triple negative breast cancer cell growth *in vitro* and *in vivo*. Thus, our study evokes the idea of treating TNBC with biocompatible α-santalol loaded chitosan NPs as an efficient drug delivery system for breast cancer therapy.

## Conflicts of interest

There are no conflicts to declare.

## Supplementary Material
